# Exploring the Anti-Osteoporotic Effects of *n*-Hexane Fraction from *Cotoneaster wilsonii* Nakai: Activation of Runx2 and Osteoblast Differentiation In Vivo

**DOI:** 10.3390/ph18010045

**Published:** 2025-01-03

**Authors:** Soyeon Hong, Hee Ju Lee, Da Seul Jung, Saruul Erdenebileg, Hoseong Hwang, Hak Cheol Kwon, Jaeyoung Kwon, Gyhye Yoo

**Affiliations:** 1 Smart Farm Research Center, Korean Institute of Science and Technology (KIST), Gangneung 25451, Republic of Korea; hongsso@kist.re.kr (S.H.); na041254@naver.com (D.S.J.); saruul98@gmail.com (S.E.); 2 Center for Natural Product Systems Biology, Korean Institute of Science and Technology, Gangneung 25451, Republic of Korea; hjlee81@kist.re.kr (H.J.L.); hoseong91@kist.re.kr (H.H.); hkwon@kist.re.kr (H.C.K.); 3 Department of Natural Product Applied Science, University of Science and Technology (UST), Daejeon 34113, Republic of Korea

**Keywords:** *Cotoneaster wilsonii* Nakai, bone formation, osteoblast differentiation, osteoporosis, phytomedicine, ovariectomized mice

## Abstract

Background: Osteoporosis is characterized by the microstructural depletion of bone tissue and decreased bone density, leading to an increased risk of fractures. *Cotoneaster wilsonii* Nakai, an endemic species of the Korean Peninsula, grows wild in Ulleungdo. In this study, we aimed to investigate the effects of *C. wilsonii* and its components on osteoporosis. Methods and Results: The alkaline phosphatase (ALP) activity of *C. wilsonii* extracts and fractions was evaluated in MC3T3-E1 pre-osteoblasts, and the *n*-hexane fraction (CWH) showed the best properties for ALP activity. The effects of the CWH on bone formation were assessed in MC3T3-E1 cells and ovariectomized mice. Biochemical assays and histological analyses focused on the signaling activation of osteoblast differentiation and osteogenic markers, such as ALP, collagen, and osterix. The CWH significantly activated TGF-β and Wnt signaling, enhancing osteoblast differentiation and bone matrix formation. Notably, CWH treatment improved micro-CT indices, such as femoral bone density, and restored serum osteocalcin levels compared to OVX controls. Conclusions: These results highlight the potential of the *C. wilsonii* Nakai *n*-hexane fraction as a promising therapeutic agent for managing osteoporosis.

## 1. Introduction

Osteoporosis is a metabolic disorder that weakens bone strength, significantly increasing the likelihood of fractures and associated mortality [1,2]. With an increase in the aging population worldwide, the incidence of osteoporosis patients and social costs therefrom is increasing every year. In addition to menopause, a variety of causes, such as alcohol consumption, aging, smoking, and decreased exercise, have been known to induce osteoporosis. Current osteoporosis treatments include the supplementation of bone components, such as phosphates and vitamin D, or hormones such as estradiol and the parathyroid hormone. However, existing treatments for osteoporosis often fail to adequately reduce fracture risk and are associated with significant side effects during prolonged use. As a result, there is a pressing need to develop new and more effective therapeutic options [3,4,5,6].

Bones are dynamic organs exhibiting constant bone formation and bone resorption, maintaining bone homeostasis [7]. Osteoblasts are bone-forming cells, and BMP [8], TGF β [9], and Wnt [10] pathways are well-known signaling inducers in osteoblast development. Osteoclasts are bone resorptive cells, and the OPG/RANKL/RANK [11] and TNF alpha [12] pathways are the major mediators of osteoclast differentiation. These pathways play a critical role in bone remodeling, and targeting them has been a focus of osteoporosis treatment mediated by osteoclast modulation. Examples include the use of RANKL inhibitors, such as denosumab, and the modulation of OPG levels to inhibit osteoclast activity [4]. When the balance between bone formation and resorption is impaired, solid bone microstructures diminish and osteoporosis occurs. At this point, two therapeutic strategies are expected: the induction of bone formation and suppression of bone resorption [13,14]. Unfortunately, current therapies only focus on the supplementation of bone components or suppression of bone resorption. Considering that osteoporosis patients receive these therapies and drugs, current therapies only retard the progression of osteoporosis by maintaining the present bone status, but do not address the recovery of bone density. In this study, we attempted to identify novel candidates with osteogenic activity that could induce bone formation.

Humans have been developing plant-derived ingredients for thousands of years. Treatments using plant-derived bioactive phytochemicals are being developed for various diseases in terms of safety and efficacy, including examples such as Genistein from soybeans, Tilianin from *Agastache rugosa*, and cinnamic acid from cinnamon bark [15,16,17]. However, there is still no cure for osteoporosis and the research is ongoing in various countries. *Cotoneaster wilsonii* Nakai, belonging to the family Rosaceae, is a rare endemic shrub that grows on Ulleungdo, a small island in Korea [16]. The genus *Cotoneaster* includes approximately 150 species, many of which have been used in traditional medicine and for various ecological and horticultural purposes. However, the research on *C. wilsonii* itself has been limited due to its relatively recent identification and its narrow geographic distribution. As a result, much of the existing research has focused on the basic characterization and propagation of this species. Biological studies on *C. wilsonii* have to date been sparse, limited primarily to simple screenings of its bioactivities, such as its ability to scavenge free radicals and inhibit nitric oxide production. Catechin is the only constituent identified in this plant to date. Nonetheless, the unique properties of *C. wilsonii* make it an intriguing candidate for further study, with the potential to uncover novel applications and benefits. Methanol extracts from the leaves of *C. wilsonii* have been reported to exhibit significant antioxidant and anti-inflammatory activities [18,19,20], suggesting that this plant may harbor valuable bioactive compounds with pharmaceutical and therapeutic relevance. Given these promising properties, it is surprising that the research into the potential anti-osteoporotic effects of *C. wilsonii* has not yet been undertaken. Exploring the bioactivity of this rare plant could contribute significant value to the field of natural product research and provide new avenues for treating osteoporosis.

The purpose of our study was to determine whether *C. wilsonii* affects bone health and what its mechanism is. We estimated the effect of *C. wilsonii* and its fractions on osteoblasts and an animal model of post-menopausal osteoporosis. Active molecules in *C. wilsonii* were also identified by chemical analysis, and their effects on osteoblast differentiation were evaluated.

## 2. Results

### 2.1. Stimulation of Osteoblastic Differentiation with the C. wilsonii n-Hexane Fraction

**Figure 1:**
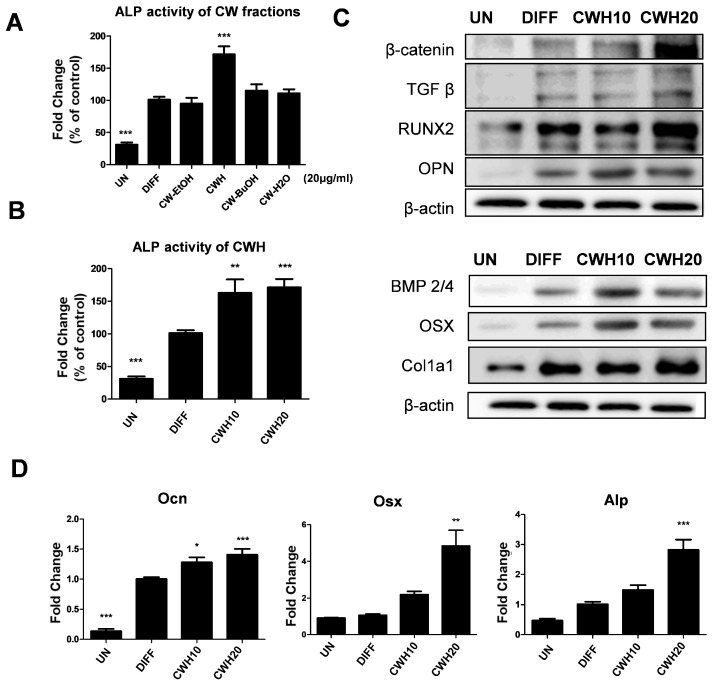
Effects of *C. wilsonii* (CW) on osteoblastic differentiation in vitro. (**A**) Comparison of ALP activity between *C. wilsonii* ethanol extract (CW-EtOH) and its fractions (CWH; *n*-Hexane fraction, CW-BuOH; buthanol fraction, CW-H2O; water-soluble layers). (**B**) ALP activity of CWH. (**C**) Protein expression analysis of osteoblastic markers using Western blot. (**D**) mRNA expression levels of osteoblastic markers determined by qRT-PCR. Data are presented as mean ± SEM (n = 6). * *p* < 0.05, ** *p* < 0.01, and *** *p* < 0.001 are significantly different from the DIFF group. UN: undifferentiated MC3T3-E1 cell; DIFF: differentiated MC3T3-E1 cell; CWH10 and CWH20: differentiated MC3T3-E1 cells treated with 10 μg/mL and 20 μg/mL CWH, respectively.

To evaluate the effects of *C. wilsonii* on osteoblast differentiation, *C. wilsonii* and its fractions were treated at a concentration of 20 μg/mL to MC3T3-E1 pre-osteoblast cells. Among the tested *C. wilsonii* ethanol extract and its three subsequent fractions, only the *n*-hexane fraction of *C. wilsonii* (CWH) demonstrated significant efficacy with an increase in alkaline phosphatase (ALP) activity, a marker for osteoblast differentiation, as shown in Figure 1A. Further dose-dependent studies revealed that CWH at concentrations between 10 and 20 μg/mL was particularly effective in enhancing ALP activity (Figure 1B). Protein analysis confirmed that CWH treatment upregulated the expression of RUNX2, a key transcription factor in osteoblast differentiation, which is mediated by the effect of CWH on the activation of the Wnt and TGF-β signaling pathways. Additionally, several downstream target proteins of RUNX2, such as OPN, OSX, and COL1A1, were also upregulated during CWH treatment (Figure 1C). mRNA analysis through RT-PCR corroborated these findings, showing that the expression levels of Ocn, Osx, and Alp are elevated by CWH treatment (Figure 1D). These results collectively suggest that the CWH fraction plays a crucial role in osteoblast differentiation through the modulation of both protein and gene expression.

### 2.2. Suppression of Post-Menopausal Osteoporosis in Ovariectomized Mice by the C. wilsonii n-Hexane Fraction

**Figure 2:**
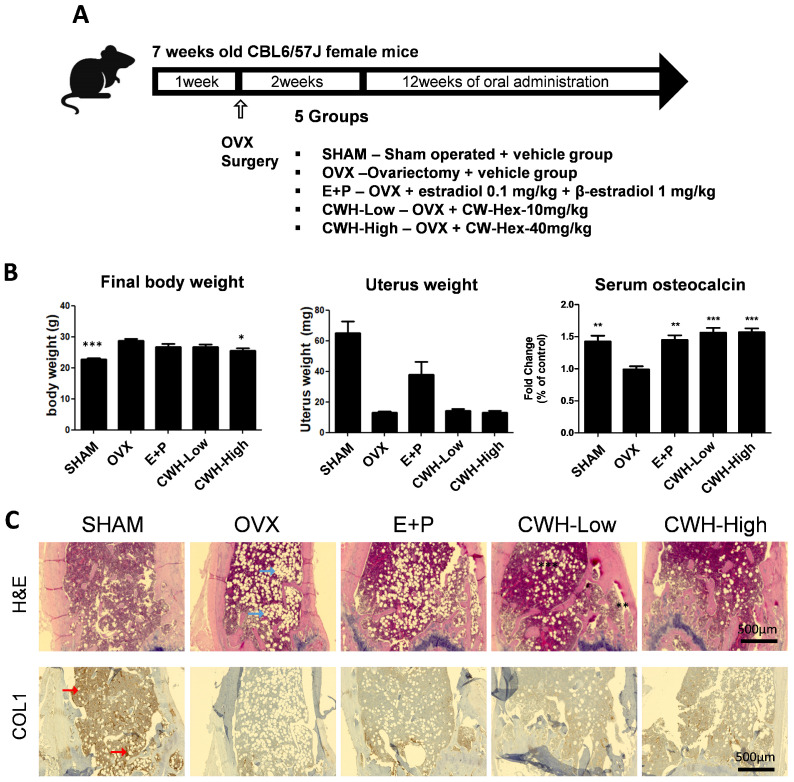
Effects of of *n*-haxane fraction of *C. wilsonii* (CWH) on physiological markers in ovariectomized mice. (**A**) Experimental design for animal study. (**B**) Measurements of body weight, uterus weight, and serum osteocalcin levels in OVX mice treated with CWH. (**C**) Histological analysis using H&E staining and type I collagen (COL1) immunohistochemical staining in the distal femoral region of OVX mice. Data are presented as mean ± SEM (n = 6). * *p* < 0.05, ** *p* < 0.01, and *** *p* < 0.001 are significantly different from the OVX group. Blue arrows: lipid droplets; Red arrows: collagen.

To investigate the potential of *C. wilsonii* to improve osteoporosis, particularly for post-menopausal bone loss, the CWH was orally administered to ovariectomized (OVX) mice, an animal model for post-menopausal osteoporosis (Figure 2A). In this model, the OVX mice demonstrated a slight increase in body weight compared to the control group (SHAM), reflecting the metabolic changes associated with estrogen deficiency. CWH treatment notably mitigated this weight gain in OVX mice significantly (Figure 2B). The weight of the uterus reduced by OVX was recovered in the E+P hormone therapy group, while it was unaffected by the CWH treatment (Figure 2B). In terms of osteoblastic activity, serum levels of osteocalcin, an osteoblast-specific marker, decreased in the OVX group, and CWH treatment significantly restored these levels, comparable to the effects observed with hormone therapy (Figure 2B). Histological analysis of H&E-stained distal femur sections revealed that an OVX-induced loss of the bone marrow structure was markedly prevented by CWH treatment (Figure 2C). Immunohistochemical staining for type I collagen indicated a reduction in collagen expression in the OVX group, which was restored by CWH treatment at a dosage of 40 mg/kg (Figure 2C).

**Figure 3:**
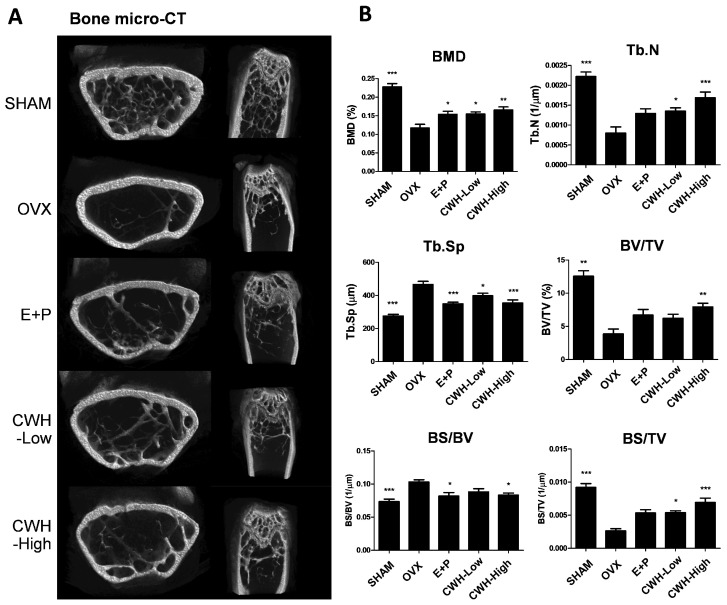
Effects of *n*-hexane fraction of *C. wilsonii* (CWH) on bone markers in ovariectomized mice. (**A**) Micro-computed tomography images of the distal femoral region of mice. (**B**) Tomographic measurements of BMD, Tb.N, BV/TV, Tb.Sp, BS/BV, and BS/TV. Data are presented as mean ± SEM (n = 6). * *p* < 0.05, ** *p* < 0.01, and *** *p* < 0.001 are significantly different from the OVX group.

Furthermore, the micro-computed tomography (micro-CT) analysis of distal femur bone architecture showed that CWH administration effectively reversed the reductions in bone density induced by OVX (Figure 3A). Specifically, the quantitative analysis demonstrated that the bone mineral density (BMD), bone surface to tissue volume ratio (BS/TV), and bone volume to tissue volume ratio (BV/TV) significantly decreased, while trabecular separation (Tb.Sp) increased in OVX mice. CWH treatment, however, significantly restored BMD, BS/TV, BV/TV, and normalized Tb.Sp values, indicating a protective effect of CWH on bone health (Figure 3B).

**Figure 4:**
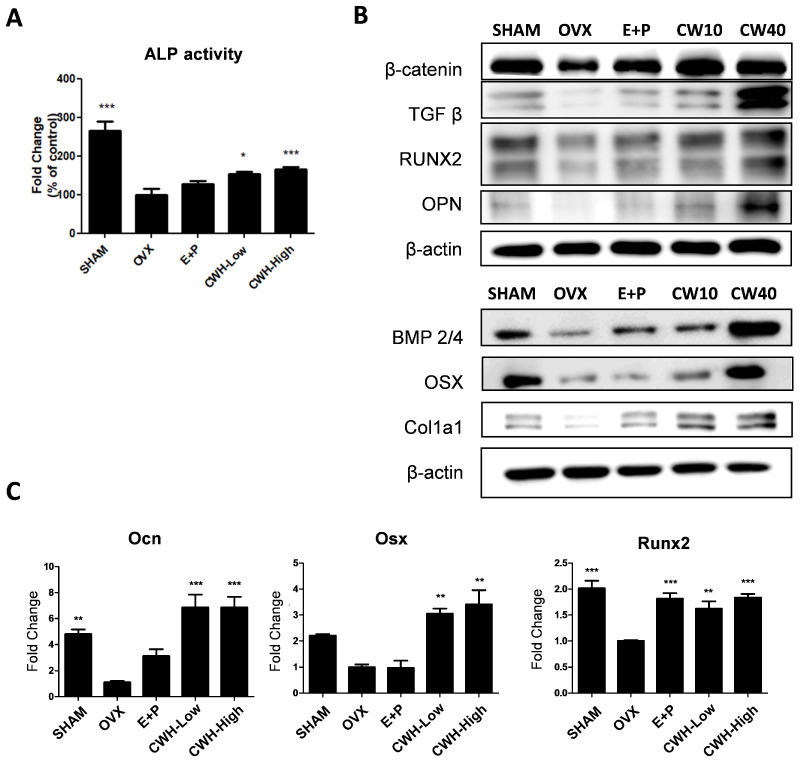
Effects *n*-hexane fraction of *C. wilsonii* (CWH) on the osteoblast population of bone marrow. (**A**) ALP activity, (**B**) protein expression, and (**C**) mRNA expression levels of osteoblastic markers in primary bone marrow cells. Data are presented as mean ± SEM (n = 6). * *p* < 0.05, ** *p* < 0.01, and *** *p* < 0.001 are significantly different from the OVX group.

To further assess the impact of the CWH on the osteoblast population and overall bone health, primary cells were extracted from the femurs of ovariectomized (OVX) mice. While ALP activity was markedly reduced in primary cells from the OVX group, a significant restoration of ALP activity was observed in primary cells from the CWH, indicating an increase in active osteoblasts (Figure 4A). When the expression levels of several crucial osteoblast markers, including RUNX2, OSX, OPN, and COL1A1, were examined, these markers were significantly down-regulated in OVX primary cells, which corresponds with the reduction in osteoblast function observed in estrogen-deficient conditions (Figure 4B). RUNX2 was notably decreased in the OVX group but significantly upregulated following CWH treatment, indicating active osteoblastogenesis. Moreover, OSX, OPN, and COL1A1—all key markers associated with bone matrix synthesis and mineralization—were similarly restored with CWH treatment. On the gene expression level, mRNA analysis revealed that genes associated with osteogenesis, such as runx2, OCN, and OSX, were significantly reduced in OVX mice, correlating with reduced bone formation. Remarkably, CWH treatment restored these mRNA levels close to those observed in SHAM control cells (Figure 4C).

### 2.3. Identification and Contents of Compounds in CWH and Its Osteoblastic Differentiation Effect

**Figure 5:**
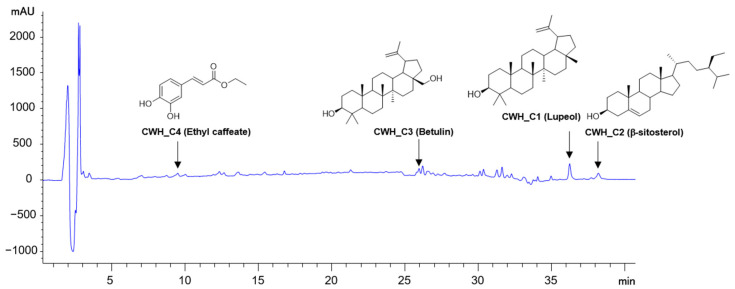
HPLC chromatogram of the *n*-hexane fraction of *C. wilsonii*. Peaks: CWH_C1, lupeol; CWH_C2, β-sitosterol; CWH_C3, betulin; CWH_C4, ethyl caffeate.

The CWH was analyzed using an HPLC system, and four compounds were identified in their HPLC chromatograms (Figure 5). The contents of four compounds in the concentration (10 mg/mL) of CWH (C4, ethyl caffeate) were 25.5 (C1, Lupeol), 9.7 (C2, β-sitosterol), 3.7 (C3, betulin), and 2.0 μg/10 mg (Figure 5). Furthermore, the structures of each compound were elucidated using NMR analysis, as shown in Supplementary Figure S1 and Table S1.

**Figure 6:**
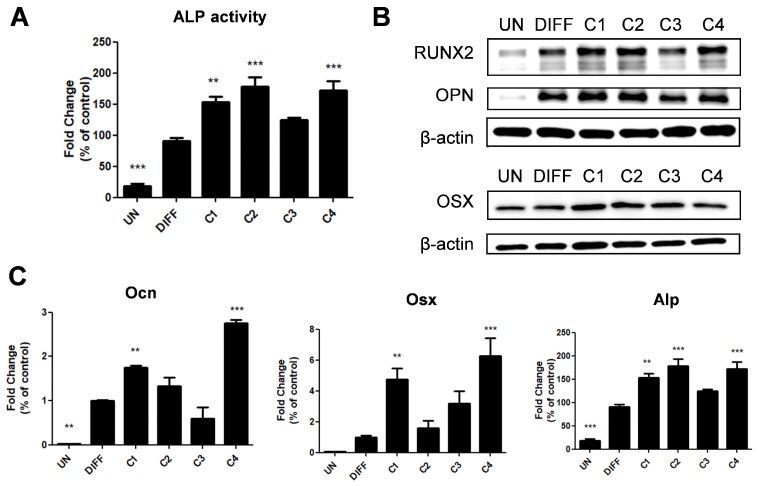
Effects of compounds of the *C. wilsonii n*-hexane fraction on osteoblastic differentiation in vitro. (**A**) ALP activity (**B**) of osteoblastic markers determined by Western blot. mRNA (**C**) expression levels of osteoblastic markers determined by qRT-PCR. Data are presented as mean ± SEM (n = 6). ** *p* < 0.01, and *** *p* < 0.001, significantly different from the DIFF group. UN: undifferentiated MC3T3-E1 cells; DIFF: differentiated MC3T3-E1 cells; C1, Lupeol; C2, β-sitosterol; C3, botulin; C4, ethyl caffeate.

This precise quantification provides insights into the bioactive components responsible for the observed osteogenic effects of CWH. The osteoblastic activity of these isolated compounds was investigated to determine their individual contributions to bone formation and differentiation. The assays revealed that three of the compounds—CWH_C1, CWH_C2, and CWH_C4—demonstrated significant efficacy in promoting ALP activity (Figure 6A). Further examinations at both the mRNA and protein expression levels were conducted to assess each compound’s role in osteoblast differentiation more precisely. However, CWH_C1, CWH_C2, and CWH_C4 exhibited particularly robust effects, as evidenced by their marked impact on osteoblast-specific gene and protein expressions (Figure 6B,C). These research results imply that CWH contains several bioactive compounds that act synergistically to promote osteoblast differentiation, and among them, CWH_C1, CWH_C2, and CWH_C4 exhibit synergistic effects and efficacy.

## 3. Discussion

Despite osteoblast differentiation and activation playing a critical role in bone homeostasis, no agents are approved by the FDA, which target osteoblasts. In our present study, we sought to find edible plants with a suppressive effect on osteoporosis via osteogenic activity and demonstrated *Cotoneaster wilsonii* Nakai had osteogenic activity in vitro and in vivo. Among the ethanol extracts of *C. wilsonii* and its fractions, only the CWH showed promotive activity in osteoblast differentiation and modulated osteogenic signaling. An animal experiment for osteoporosis with an ovariectomy confirmed osteogenic activity, where the oral administration of 10–40 mg/kg CWH treatment suppressed bone loss with the induction of osteoblast differentiation. These data suggest CWH treatment reduces bone loss during osteoporosis progression, and its effect on the bone resulted from the osteogenic property of CWH.

When osteoblasts are differentiated, osteogenic signaling is activated and modulates osteoblast differentiation. RUNX2 is one of representative osteogenic markers [21,22,23]. Because RUNX2 functions as a transcription factor, its action alters gene expressions related to osteogenic signaling, such as COL1Al, ALP, and OCN. Our data show an increase in RUNX2 in vitro and ex vivo, which subsequently results in the elevation of ALP activity and COL1Al expression. These molecules enhance the extracellular matrix and calcium–phosphate complex to induce osteoid mineralization. Because OCN plays a pivotal role in apatite crystallization, OCN is a major indicator used to estimate bone health in the clinical study. In our study, RUNX2’s increase with CWH treatment elevated OCN levels in the serum and the bone. These data indicate that CWH shows osteogenic activity, resulting in a higher active osteoblast population in mice femurs. COL1A1 is a component of osteoids, which become a calcified bone matrix with hydroxyapatite [24,25]. Our data show CWH elevates COL1A1 in vitro, ex vivo, as well as in bone sections. These data indicate CWH elevates the bone formation process by its osteogenic activity on osteoblast differentiation, resulting in reduced bone loss during estrogen deficiency. Therefore, we can conclude that CWH has an osteogenic property and its effect can improve the bone status in post-menopausal osteoporosis animal models.

As mentioned previously, in addition to hormones, Wnt, BMP, and TGFβ signaling are major modulators in the differentiation of osteoblasts [8,9,10]. Each signaling type has been well studied and does not exist independently, but is organically connected through crosstalk [26]. Wnt signaling is one of the most important signaling types in cell proliferation and differentiation, including osteogenesis in mesenchymal stem cells. Wnt signaling is divided into canonical and non-canonical pathways, and the canonical pathway mediated by β-catenin has been actively studied as a target for osteoporosis [27]. In the presence of Wnt ligands, β-catenin regulates the expression of target genes, such as RUNX2 and OSX. TGFβ signaling is a well-known pathway regulating immune responses, and this signaling on bone mineralization has been well studied with the isoforms of TGFβ knockout mice. Among these, TGF beta1 has been studied to be mainly involved in the activation of osteoblasts [28]. Previous studies suggest that Wnt and TGFβ1 signaling regulate osteoblast differentiation through the modulation of RUNX2, which alters the gene expression of target molecules, collagen, OSX, and OCN [29]. These target molecules are representative osteogenic makers regulating skeletal development, cartilage formation, bone formation, and bone homeostasis. Our data show the effect of CWH on Wnt and TGFβ1 signaling, and osteogenic signaling, including RUNX2, collagen, OSX, and OCN. This change by the CWH resulted in a positive effect on bone density analysis through micro-CT and other tests. Therefore, we can conclude that CWH has suppressive activity on post-menopausal osteoporosis via the induction of Wnt and TGFβ1 signaling. Still, further study is required to clarify how CWH modulates signaling.

Chemical analysis revealed the presence of four major bioactive phytocompounds in the CWH fraction: lupeol, β-sitosterol, betulin, and ethyl caffeate. Lupeol, a triterpenoid, has been shown to exhibit anti-inflammatory, antioxidant, and anti-alcohol effects. β-sitosterol, a phytosterol commonly extracted from plant oils such as soy and corn, is well-documented for its anti-cholesterolemic, anti-inflammatory, and anti-diabetic properties [30,31,32]. Betulin has demonstrated anti-inflammatory, antiviral, antibacterial, and osteoclast-inhibitory effects, while ethyl caffeate is recognized for its antioxidant, anti-inflammatory, and skin carcinogenesis-inhibitory activities [33,34,35,36,37]. Previous research has highlighted the role of lupeol in restoring bone loss in hypercalcemic rats by inhibiting osteoclast differentiation and β-sitosterol in reversing dexamethasone-induced bone damage through its antioxidant effects [38,39]. Despite these findings, the osteoblast-specific effects of these compounds have not been explored until now. Our study is the first to report their osteogenic activity. However, since the osteogenic effects of the individual compounds were not as pronounced as those of the CWH fraction, it is likely that their combined action leads to a synergistic effect, enhancing the overall bioactivity of CWH.

A limitation of this study is that we did not identify the direct molecular or microbial targets of the fractions or individual compounds. Although we successfully demonstrated the osteogenic potential of CWH and its bioactive components, further studies are needed to uncover the precise mechanisms underlying these effects. Investigating protein targets, signaling interactions, or potential microbiome contributions could offer deeper insights into how these compounds promote bone health. Future research utilizing advanced techniques, such as proteomics, molecular docking, or microbiome analysis, is recommended to fill this gap and further validate the therapeutic potential of *Cotoneaster wilsonii* extracts.

## 4. Materials and Methods

### 4.1. Plant Materials

The seeds of *Cotoneaster wilsonii* were collected in Ulleungdo in May 1992, multiplied and planted in Key-chungsan Botanical Garden, and then distributed to Hantaek Botanical Garden. Branches and leaves (HTS2020-0063) were collected in June 2020, dried, and ground.

### 4.2. Extraction and Isolation of Compounds 1−4 from C. wilsonill HPLC Analysis

The dried plant (1.18 kg) was extracted with 70% EtOH/water (2 × 12 L, Duksan Pure Chemicals, Ansan, Republic of Korea) at 25 °C for 7 days and evaporated under reduced pressure to obtain a crude extract (192.6 g). *C. wilsonii* extract was suspended in water and partitioned in turn with *n*-hexane (3 × 450 mL, Duksan Pure Chemicals, Ansan, Republic of Korea) and *n*-BuOH (2 × 450 mL, Duksan Pure Chemicals, Ansan, Republic of Korea) to yield *n*-hexane-soluble (9.8 g), *n*-BuOH-soluble (57.2 g), and water-soluble layers. The *n*-hexane fraction (7 g) was chromatographed on a Silica gel column (Merck, Darmstadt, Germany), eluting with hexane–ethyl acetate (15:1→1:1, *v*/*v*, Duksan Pure Chemicals, Ansan, Republic of Korea) to produce fourteen fractions (fractions 1–14). The content was determined by comparing the peak area under the concentration (1 mg/mL) of each compound. The relative concentrations of these compounds were determined by comparing their chromatogram peak areas with those of the reference standards at a concentration of 1 mg/mL for each compound. Compound 1 (5.2 mg) was re-chromatographed from fraction 5 using the Silica gel [Hexane-Ethyl acetate (5:1, *v*/*v*)] column chromatography. From fraction 7, compound 2 (7.3 mg) was isolated with the Silica gel [Hexane-Ethyl acetate (5:1→1:1, *v*/*v*)] column chromatograph to obtain white crystals. Fraction 9 was purified using Silica gel [Hexane-Ethyl acetate (6:1→4:1, *v*/*v*)] column chromatography to obtain sub-fractions 9.1−9.5. Compound 3 (9.3 mg) was isolated using Silica gel [Hexane-Ethyl acetate (5:1→3:1, *v*/*v*)] from fraction 9.4. Fraction 11 was separated by Sephadex LH-20 with MeOH (Daejung Chemicals & Metals Co., Ltd., Siheung, Republic of Korea) and fraction 11.3 was finally purified by semi-preparative HPLC using a Phenomenex Luna C18 column (20 × 250 mm, 5 μm, Phenomenex, Torrance, CA, USA) with 25% isocratic MeCN (26 min, 3 mL/min, 254 nm) to yield compound 4 (6.2 mg). The chemical structures of four compounds were determined by 1H and 13C nuclear magnetic resonance data and compared with the reported data [40,41,42,43].

### 4.3. MC3T3-E1 Mouse Pre-Osteoblast Cell Culture

MC3T3-E1 mouse pre-osteoblast cells (ATCC, Manassas, VA, USA) were maintained in α-minimum essential medium (α-MEM, Gibco, Gaithersburg, MD, USA) supplemented with fetal bovine serum (Gibco) and 1% penicillin–streptomycin (P/S, Gibco) in a 5% CO_2_ incubator at 37 °C. To induce osteoblastic activation, the cells were cultured in differentiation medium (DM) containing 50 μg/mL L-ascorbic acid (Sigma Aldrich, St. Louis, MO, USA ) and 10 mM β-glycerophosphate (Sigma Aldrich, St. Louis, MO, USA ).

### 4.4. Alkaline Phosphatase Activity

After 6 days of differentiation with DM, the cells were lysed using 0.1% Triton-X-100 lysis buffer. Alkaline phosphatase activity was quantified at 405 nm using the SensoLyte pNPP Alkaline Phosphatase Assay Kit (AnaSpec, Fremont, CA, USA).

### 4.5. Western Blotting and RT-PCR

Protein analysis was conducted using Western blotting, following previously described protocols [22,23]. Primary antibodies targeting β-actin, RUNX2 (Runt-related transcription factor 2), Osteopontin (OPN), Osterix (OSX), bone morphogenetic proteins 2 and 4 (BMP2/4), transforming growth factor β (TGFβ), collagen type 1 alpha 1 (Col1a1), along with secondary anti-mouse and anti-rabbit antibodies, were obtained from Santa Cruz Biotechnology (Dallas, TX, USA). Antibodies specific to β-catenin were procured from Cell Signaling Technology (Danvers, MA, USA). For mRNA analysis, qRT-PCR was employed based on previously published methods. Relative mRNA levels were quantified using specific primers and normalized to GAPDH expression as the internal control. Primer sequences used for the analysis are listed in Table 1.

### 4.6. Animal Experiment

Animal studies were performed as outlined in our prior research [22,23]. Eight-week-old female C57BL/6J mice (Central Lab. Animal Inc., Seoul, Republic of Korea) were subjected to an ovariectomy (OVX) under anesthesia. The experimental groups included a SHAM-operated group (n = 8) and a treatment group receiving the *C. wilsonii n*-hexane fraction (CWH) at 40 mg/kg (n = 8). The dosages for CWH (10 mg/kg and 40 mg/kg) were determined based on prior in vitro experiments, while the concentrations for E+P (0.1 mg/kg β-estradiol and 1 mg/kg progesterone, Sigma Aldrich, St. Louis, MO, USA) were derived from previous studies [16,17]. CWH and E+P solutions were prepared in a 0.5% carboxymethyl cellulose (CMC) solution (Sigma Aldrich, St. Louis, MO, USA) containing 1% sesame oil and 0.5% DMSO (Sigma Aldrich, St. Louis, MO, USA). Similarly, SHAM and OVX groups received a 0.5% CMC solution containing 1% sesame oil and 0.5% DMSO.

After 12 weeks of oral administration (14 weeks post-OVX surgery), blood samples, uteri, and femurs were collected for subsequent analyses. The 14-week OVX period was chosen based on preliminary findings showing consistent changes in bone microstructural markers that were not apparent at 12 weeks but became clear by 14 weeks [16,17,44]. All procedures with the potential to cause pain were conducted under appropriate anesthesia. Surgeries were performed using inhalation anesthesia, and dissections were completed under anesthetic injection. Blood was collected via cardiac puncture while the mice remained anesthetized. All animal care and procedures adhered to ethical guidelines and were approved by the Animal Use and Care Committee of the Korea Institute of Science and Technology (KIST-2021-01-003), Gangneung, Republic of Korea.

### 4.7. Mouse Osteoblast Primary Cell Culture

Right femurs were harvested from mice 14 weeks post-operation and maintained in PBS (Welgene, Gyeongsan, Republic of Korea) at room temperature for primary cell isolation. The detailed procedure for isolating and culturing primary cells was performed based on established methods [16,17,45]. Bone marrow cells were extracted by flushing with PBS, while adherent cells from the bone were digested using collagenase (Sigma Aldrich, St. Louis, MO, USA). The collagenase-digested cells were collected through centrifugation. These isolated cells were then cultured on collagen-coated plates in α-MEM supplemented with 10% fetal calf serum (Gibco, Waltham, MA, USA) and 1% penicillin–streptomycin (P/S, Cytiva, Marlborough, MA, USA) at 37 °C in a 5% CO_2_ incubator for 24 h. Cells at passage 2–3 were treated with differentiation medium (DM) for 6 days prior to analysis.

### 4.8. Micro-Computed Tomography Plant Materials

Left femurs were fixed in formalin (Sigma Aldrich, St. Louis, MO, USA) for micro-CT and histological evaluations. Distal femurs were scanned using the SkyScan 1172 micro-CT system (Bruker MicroCT Corp., Kontich, Belgium). The scanned data were processed using Nrecon software v1.7.3.2 and CTAn integrated software v.1.17.7.2 software (Bruker MicroCT Corp.) to measure bone mineral density (BMD), bone surface area/bone volume (BS/BV), trabecular spacing (Tb.Sp), trabecular number (Tb.N), bone surface area/total volume (BS/TV), and bone volume/total volume (BV/TV).

### 4.9. Hematoxylin–Eosin Staining (H&E) and Immunohistochemistry (IHC)

After the micro-CT analysis, the left distal femurs were fixed in formalin and prepared for the histological examination. Paraffin-embedded femoral bone sections were cut to a thickness of 5 μm and stained with hematoxylin and eosin (H&E) as well as the collagen type I antibody (Abcam, Cambridge, UK).

### 4.10. Serum Parameters

Serum osteocalcin (OCN) levels were quantified using the Gla-type osteocalcin (Gla-OC) EIA Kit (TaKaRa, Tokyo, Japan). Blood samples were centrifuged at 14,000 rpm for 30 min at 4 °C. Ten microliters of serum were diluted 10-fold and used for analysis.

### 4.11. HPLC Analysis of n-Hexane Fraction from C. wilsonii

High-performance liquid chromatography (HPLC) analysis was performed using the Agilent 1200 series HPLC system (Agilent Technologies, Santa Clara, CA, USA) with a Phenomenex Luna C8(2) column (4.6 × 150 mm, 5 μm). The mobile phase consisted of 0.1% formic acid (Sigma Aldrich, St. Louis, MO, USA) in water (A) and 0.1% formic acid in acetonitrile (Fisher Scientific, Fair Lawn, NJ, USA) (B) with the following gradients: 0–3 min, isocratic at 30% B; 3–30 min, linear gradient from 30% to 100% B; 30–40 min, isocratic at 100% B; and 40–50 min, return to initial conditions. The flow rate was 0.8 mL/min with an injection volume of 10 μL. Detection was conducted at 210 nm.

### 4.12. Statistical Analysis

All experiments were performed in triplicate or included all mice within each group, with each procedure repeated three times for consistency. Data are expressed as mean ± SEM. Statistical analysis was conducted using one-way ANOVA with SPSS 17.0 and GraphPad Prism 7.0 software. Duncan’s multiple range test was used for post hoc comparisons. Statistical significance was determined at *p* < 0.05.

## 5. Conclusions

*C. wilsonii* is a unique plant species endemic to Korea, with no prior research exploring its effects on bone health. In this study, we demonstrated that the *n*-hexane fraction of *C. wilsonii* (CWH) exhibits strong potential to induce osteoblast differentiation. Our findings reveal that CWH influences key osteogenic signaling pathways, including RUNX2 activation mediated by Wnt and TGF-β pathways. These molecular interactions significantly promoted bone formation, which led to enhanced bone density and offered protection against ovariectomy-induced bone loss in our experimental models. The observed osteogenic effects of CWH were partly attributable to its four primary bioactive compounds: lupeol, β-sitosterol, betulin, and ethyl caffeate. Each of these compounds is known for its diverse biological properties, including anti-inflammatory and antioxidant activities. While their individual effects on bone health via osteoclast suppression have been documented, this study highlights the synergistic osteogenic contribution of these compounds within the CWH fraction, amplifying its therapeutic efficacy. Our findings underscore the potential of CWH as a promising natural anti-osteoporotic agent with the advantage of potentially fewer side effects compared to conventional treatments.

## Figures and Tables

**Table 1:** Sequences of PCR primers.

Gene	Forward (5′–3′)	Reverse (5′–3′)
Alp	GATCATTCCCACGTTTTCAC	TGCGGGCTTGTGGGACCTGC
Ocn	AGACTCCGGCGCTACCTT	CTCGTCACAAGCAGGGTTAAG
Osx	TGAGGAAGAAGCCCATTCAC	ACTTCTTCTCCCGGGTGTG
Runx2	TCCACAAGGACAGAGTCAGATTAC	TGGCTCAGATAGGAGGGGTA
Gapdh	AAGAGGGATGCTGCCCTTAC	CCATTTTGTCTACGGGACGA

## Data Availability

The original contributions presented in the study are included in the article, and further inquiries can be directed to the corresponding authors.

## Supplementary Materials

The following supporting information can be downloaded at: https://www.mdpi.com/article/10.3390/ph18010045/s1, Figure S1: H (500 MHz) and 13C (125 MHz) NMR spectra of compounds 1−4 from *C. wilsonii*. (A and B; Compound 1, Pyridine-d5, C and D; Compound 2, Pyridine-d5, E and F; Compound 3, Pyridine-d5, G and H; Compound 4, CD3OD); Table S1: 1H and 13C NMR data of compounds 1−4.

## References

[1] Bliuc D., Alarkawi D., Nguyen T.V., Eisman J.A., Center J.R. (2015). Risk of subsequent fractures and mortality in elderly women and men with fragility fractures with and without osteoporotic bone density: The Dubbo Osteoporosis Epidemiology Study. J. Bone. Miner. Res..

[2] Lin J.T., Lane J.M. (2004). Osteoporosis: A Review. Clin. Orthop. Relat. Res..

[3] Van den Bergh J.P., van Geel T.A., Geusens P.P. (2012). Osteoporosis, frailty and fracture: Implications for case finding and therapy. Nat. Rev. Rheumatol..

[4] Kendler D.L., Cosman F., Stad R.K., Ferrari S. (2022). Denosumab in the treatment of osteoporosis: 10 years later: A narrative review. Adv. Ther..

[5] Skjødt M.K., Frost M., Abrahamsen B. (2019). Side effects of drugs for osteoporosis and metastatic bone disease. Br. J. Clin. Pharmacol..

[6] Martiniakova M., Babikova M., Omelka R. (2020). Pharmacological agents and natural compounds: Available treatments for osteoporosis. J. Physiol. Pharmacol..

[7] Gao Y., Patil S., Jia J. (2021). Development of molecular biology of osteoporosis. Int. J. Mol. Sci..

[8] (2006). BMP signaling is required for RUNX2-dependent induction of the osteoblast phenotype. J. Bone Miner. Res..

[9] Chen G., Deng C., Li Y.-P. (2012). TGF-β and BMP signaling in osteoblast differentiation and bone formation. Int. J. Biol. Sci..

[10] Kim J.H., Liu X., Wang J., Chen X., Zhang H., Kim S.H., Cui J., Li R., Zhang W., Kong Y. (2013). Wnt signaling in bone formation and its therapeutic potential for bone diseases. Ther. Adv. Musculoskelet. Dis..

[11] Hofbauer L.C., Kuhne C.A., Viereck V. (2004). The OPG/RANKL/RANK system in metabolic bone diseases. J. Musculoskelet. Neuronal Interact..

[12] Zha L., He L., Liang Y., Qin H., Yu B., Chang L., Xue L. (2018). TNF-α contributes to postmenopausal osteoporosis by synergistically promoting RANKL-induced osteoclast formation. Biomed. Pharmacother..

[13] Henriksen K., Bollerslev J., Everts V., Karsdal M.A. (2011). Osteoclast activity and subtypes as a function of physiology and pathology—Implications for future treatments of osteoporosis. Endocr. Rev..

[14] Henriksen K., Tanko L.B., Qvist P., Delmas P.D., Christiansen C., Karsdal M.A. (2007). Assessment of osteoclast number and function: Application in the development of new and improved treatment modalities for bone diseases. Osteoporos. Int..

[15] Pereira J.V., Modesto-Filho J., de FAgra M., Barbosa-Filho J.M. (2002). Plant and plant-derived compounds employed in prevention of the osteoporosis. Acta Farm. Bonaer..

[16] Hong S., Cha K.H., Hye Park J., Choi J.H., Yoo G., Nho C.W. (2022). Cinnamic acid suppresses bone loss via induction of osteoblast differentiation with alteration of gut microbiota. J. Nutr. Biochem..

[17] Hong S., Cha K.H., Son Y.J., Kim S.M., Choi J.H., Yoo G., Nho C.W. (2021). *Agastache rugosa* ethanol extract suppresses bone loss via induction of osteoblast differentiation with alteration of gut microbiota. Phytomedicine.

[18] Chang C.S., Jeon J.I. (2003). Leaf flavonoids in *Cotoneaster wilsonii* (Rosaceae) from the island Ulleung-do, Korea. Biochem. Syst. Ecol..

[19] Yoo N.H., Kim H.K., Lee C.O., Park J.H., Kim M.J. (2019). Comparison of anti-oxidant and anti-inflammatory activities of methanolic extracts obtained from different parts of *Cotoneaster wilsonii* Nakai. Korean J. Med. Crop Sci..

[20] Yoo N.H., Kim H.K., Song J.M., Lee C.O., Park J.H., Park B.J., Choi Y.B., Baek Y.S., Hwang Y.J., Kim M.J. (2019). Biological Activities and Separation of Active Substance of Extract and Fractions from *Cotoneaster wilsonii* Nakai Leaf. Korean J. Med. Crop Sci..

[21] Vimalraj S., Arumugam B., Miranda P.J., Selvamurugan N. (2015). Runx2: Structure, function, and phosphorylation in osteoblast differentiation. Int. J. Biol. Macromol..

[22] Liu T.M., Lee E.H. (2013). Transcriptional regulatory cascades in Runx2-dependent osteogenic differentiation. Tissue Eng. Part B Rev..

[23] Karsenty G. (2019). Update on the roles of Runx2 in skeletal biology. J. Bone Miner. Res..

[24] Dirckx N., Van Hul M., Maes C. (2013). Osteoblast recruitment to sites of bone formation in skeletal development, homeostasis, and regeneration. Birth Defects Res. Part C Embryo Today Rev..

[25] Reffitt D.M., Ogston N., Jugdaohsingh R., Cheung H.F.J., Evans B.A.J., Thompson R.P.H., Powell J.J., Hampson G.N. (2003). Orthosilicic acid stimulates collagen type 1 synthesis and osteoblastic differentiation in human osteoblast-like cells in vitro. Bone.

[26] Guo X., Wang X.F. (2009). Signaling cross-talk between TGF-β/BMP and other pathways. Cell Res..

[27] Glass D.A., Karsenty G. (2006). Molecular bases of the regulation of bone remodeling by the canonical Wnt signaling pathway. Curr. Top. Dev. Biol..

[28] Wu M., Chen G., Li Y.P. (2016). TGF-β and BMP signaling in osteoblast, skeletal development, and bone formation, homeostasis and disease. Bone Res..

[29] Jing Z., Liang Z., Yang L., Du W., Yu T., Tang H., Li C., Wei W. (2022). Bone formation and bone repair: The roles and crosstalk of osteoinductive signaling pathways. Process Biochem..

[30] Paniagua-Pérez R., Flores-Mondragón G., Reyes-Legorreta C., Herrera-López B., Cervantes-Hernández I., Madrigal-Santillán O., Moralez-González J.A., Álvarez-González I., Madrigal-Bujaidar E. (2017). Evaluation of the anti-inflammatory capacity of beta-sitosterol in rodent assays. Afr. J. Tradit. Complement. Altern. Med..

[31] Ikeda I., Sugano M. (1983). Some aspects of mechanism of inhibition of cholesterol absorption by β-sitosterol. Biochim. Biophys. Acta Biomembr..

[32] Babu S., Jayaraman S. (2020). An update on β-sitosterol: A potential herbal nutraceutical for diabetic management. Biomed. Pharmacother..

[33] Wang T., Li S., Yi C., Wang X., Han X. (2022). Protective Role of β-Sitosterol in Glucocorticoid-Induced Osteoporosis in Rats Via the RANKL/OPG Pathway. Altern. Ther. Health Med..

[34] Liu Y., Xue Y. (2023). Study on the Mechanism of Beta-Sitosterol Involved in the Regulation of Senile Postmenopausal Osteoporosis through PI3K-Akt Signal Pathway. Indian J. Pharm. Sci..

[35] Ci X., Zhou J., Lv H., Yu Q., Peng L., Hua S. (2017). Betulin exhibits anti-inflammatory activity in LPS-stimulated macrophages and endotoxin-shocked mice through an AMPK/AKT/Nrf2-dependent mechanism. Cell Death Dis..

[36] Virginia F., Cathrine L., Fernandez S. (2024). Isolation, purification, and characterization of betulin, a pentacyclic triterpenoid from the ethanolic extracts of *Coleus forskohlii* tuberous roots; its antimicrobial, antioxidant, anti-inflammatory and cytotoxicity appraisals: Phytochemistry. Moroc. J. Chem..

[37] Kim K.J., Lee Y., Hwang H.G., Sung S.H., Lee M., Son Y.J. (2018). Betulin suppresses osteoclast formation via down-regulating NFATc1. J. Clin. Med..

[38] Im N.K., Lee D.S., Lee S.R., Jeong G.S. (2016). Lupeol isolated from Sorbus commixta suppresses 1α, 25-(OH) 2D3-mediated osteoclast differentiation and bone loss in vitro and in vivo. J. Nat. Prod..

[39] Chauhan S., Sharma A., Upadhyay N.K., Singh G., Lal U.R., Goyal R. (2018). In-vitro osteoblast proliferation and in-vivo anti-osteoporotic activity of *Bombax ceiba* with quantification of Lupeol, gallic acid and β-sitosterol by HPTLC and HPLC. BMC Complement. Altern. Med..

[40] Maciel e Silva A.T., Gonçalves Magalhães C., Pains Duarte L., da Nova Mussel W., Gois Ruiz A.L.T., Shiozawa L., de Carvalho J.E., Trindade I.C., Vieira Filho S.A. (2017). Lupeol and its esters: NMR, powder XRD data and in vitro evaluation of cancer cell growth. Braz. J. Pharm. Sci..

[41] Credo D., Mabiki F.P., Machumi F., Cornett C. (2022). Structural elucidation and toxicity evaluation of bioactive compounds from the leaves and stem woods *of Synadenium glaucescens* pax. Pharm. Sci. Res..

[42] Patra A., Chaudhuri S.K., Panda S.K. (1988). Betulin-3-caffeate from *Quercus suber*, 13C-nmr spectra of some lupenes. J. Nat. Prod..

[43] Xiang M., Su H., Hu J., Yan Y. (2011). Isolation, identification and determination of methyl caffeate, ethyl caffeate and other phenolic compounds from *Polygonum amplexicaule* var. sinense. J. Med. Plants Res..

[44] Park J.H., Son Y.J., Lee C.H., Nho C.W., Yoo G. (2020). *Circaea mollis* Siebold & Zucc. Alleviates postmenopausal osteoporosis in a mouse model via the BMP-2/4/Runx2 pathway. BMC Complement. Med. Ther..

[45] Soleimani M., Nadri S. (2009). A protocol for isolation and culture of mesenchymal stem cells from mouse bone marrow. Nat. Protoc..

