# Supplementary material for: Exploring the Anti-Osteoporotic Effects of n-Hexane Fraction from Cotoneaster wilsonii Nakai: Activation of Runx2 and Osteoblast Differentiation In Vivo
Source: Pharmaceuticals (Basel). 2025 Jan 3;18(1):45. doi: 10.3390/ph18010045 (PMC11768920; doi:10.3390/ph18010045)
Supplement: Supplementary file 1 [file pharmaceuticals-18-00045-s001.zip › pharmaceuticals-3351757-supplementary.pdf]

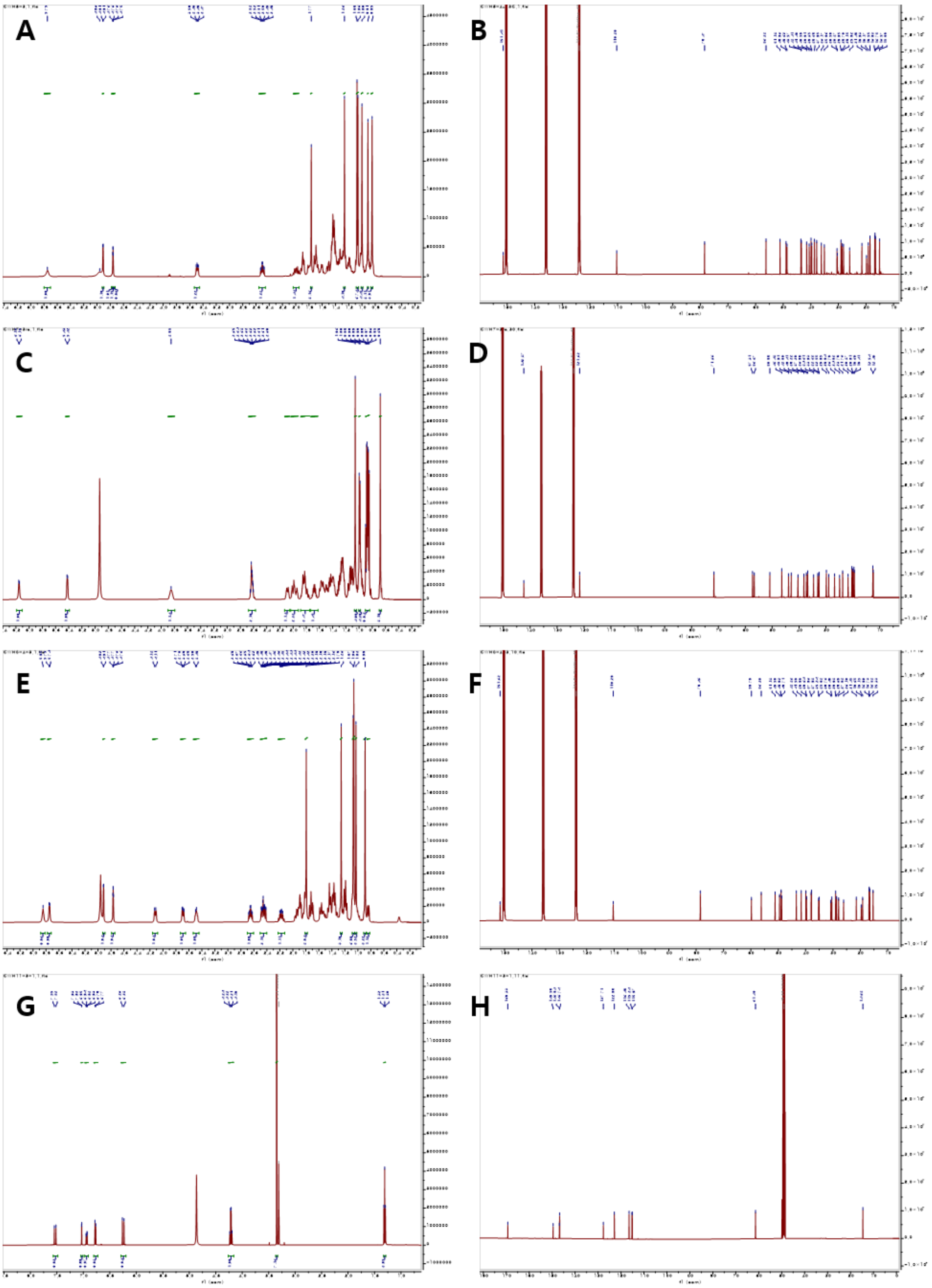

Supplementary Figure S1. ^{1}H (500 MHz) and ^{13}C (125 MHz) NMR spectra of compounds **1** −**4** from *C. wilsonii.* (A and B; Compound **1**, Pyridine-d_5, C and D; Compound **2**, Pyridine-d_5, E and F; Compound **3**, Pyridine-d_5, G and H; Compound **4**, CD_3OD)

Supplementary Table S1. ^{1}H and ^{13}C NMR data of compounds **1**−**4**

no	**1**		**2**		**3**		**4**	
	δ_H (multi, *J* in Hz)	δ_C	δ_H (multi, *J* in Hz)	δ_C	δ_H (multi, *J* in Hz)	δ_C	δ_H (multi, *J* in Hz)	δ_C
1		39.6		38.2		39.6		127.7
2		28.7		32.6		28.7		122.9
3	3.49 (dd, 10.1, 6.2)	78.5	3.85 (br s)	71.7	3.48 (m)	78.5	6.77 (d, 8.2)	116.5
4		39.9		42.9		39.9	6.94 (dd, 8.2, 2.1)	146.7
5		56.2		142.4		55.2		149.6
6		19.2	5.42 (d, 4.2)	121.6		19.2	7.03 (d, 2.1)	115.1
7		35.1		33.0		35.2	7.53 (d, 15.9)	146.8
8		41.5		32.6		41.6	6.25 (d, 15.9)	115.2
9		51.1		50.9		51.1		169.3
10		37.9		37.3		37.8	4.21 (q, 7.1)	61.4
11		21.5		21.8		21.5	1.31 (t, 7.1)	14.6
12		25.9		40.4		26.1		
13		38.7		43.9		38.0		
14		43.4		57.3		43.4		
15		28.7		24.9		27.9		
16		36.2		28.9		30.4		
17		43.6		56.7		48.7		
18		48.9		12.4		49.5		
19	2.50 (td, 11.0, 5.9)	48.6		20.4		48.9		
20		151.4		36.8		151.6		
21		30.4	0.68 (s)	19.4		30.8		
22		40.6	0.85 (d, 6.8)	34.6		35.0		
23	1.08 (s)	29.1	0.87 (d, 6.8)	25.8	1.05 (s)	29.0		
24	0.85 (s)	16.6	0.89 (overlapped)	46.4	0.89 (s)	16.5		
25	1.00 (s)	16.8	0.99 (d, 6.4)	29.9	1.03 (s)	16.7		
26	1.26 (s)	16.8	1.06 (s)	20.0	1.07 (s)	16.8		
27	1.06 (s)	15.1		19.6	1.26 (s)	15.3		
28	0.91 (s)	18.6		23.8	4.12 (d, 9.3), 3.69 (dd, 10.7, 3.6)	59.8		
29	4.91 (d, 2.6), 4.76 (dd, 2.6, 1.4)	110.3		12.5	4.91 (d, 2.6), 4.77 (dd, 2.6, 1.4)	110.3		
30	1.77 (s)	19.8			1.79 (s)	19.6		
